# 

**Published:** 1965-01

**Authors:** 


### BRISTOL MEDICO-CHIRURGICAL JOURNAL

*(Incorporating the Medical Journal of the South-West)*

Established 1883

VOL. 80 (i) January 1965 NO. 295

### Contents

ATAXIA AND HYPOTHYROIDISM. . . *B. Ashworth* 1

CHROMOSOME ABNORMALITY AS A POSSIBLE CAUSE OF HABITUAL ABORTION . . . . *L. Wingate* 5

TRANSLOCATION MONGOLISM ARISING DE NOVO?. . . . . . . . . *P. W. Short* 7

ARHINENCEPHALY — A SHORT REVIEW *T. F. Draisey, et al.* 13

APPOINTMENTS . . . . . . . . . 17

*PUBLISHED QUARTERLY*

*ANNUAL SUBSCRIPTION* 16/- *POST FREE*

BRISTOL MEDICO-CHURURGICAL JOURNAL

PUBLISHED UNDER THE AUSPICES OF THE

### Bristol Medico-Chirurgical Society

The Society was founded in 1874. In 1883 publication of this Journal began, and has continued quarterly since then. During the years 1949 to 1962 it appeared under the title of "The Medical Journal of the South West".

The Society founded a medical library in 1890, which in 1892 was combined with that of the Bristol Medical School. This became the University of Bristol Medical Library in 1925, and an agreement in that year confirmed the privilege of Members to use the University Medical Library.

### Editorial Committee

Dr. N. J. Brown, Southmead Hospital, Bristol. *Joint Editor.*

Dr. A. B. Raper, Department of Pathology, Bristol Royal Infirmary. *Joint Editor.*

Dr. J. Macrae, Ham Green Hospital.

Dr. R. C. Wofinden, Central Clinic, Bristol.

Mr. A. E. S. Roberts, Medical Library, University of Bristol. *Secretary.*

*Local Correspondents*

*Bath* . . Mr. A. D. Bateman, 3 The Circus, Bath.

*Exeter* . . Dr. L. N. Jackson, Mount Jocelyn, Crediton, Devon.

*Gloucester* . Dr. R. F. Jarrett, 9 College Green, Gloucester.

*Plymouth* . Dr. C. R. Croft, Lexden, Hartley Av., Plymouth.

*Taunton* . Dr. M. McAlley, 1 The Crescent, Taunton.

*Torquay* . Dr. A. Robinson Thomas, 20 Devon Square, Newton Abbot, Devon.

*Truro* . . Dr. F. D. M. Hocking, St. Andrews, Perranporth, Cornwall.

*Yeovil* . . Mr. J. A. Vere Nicoll, Knapp House, Yeovil, Somerset.

### NOTICE TO CONTRIBUTORS

Original articles are invited, provided they have not been published elsewhere. Notes of forthcoming events, meetings held, degrees conferred, staff appointments, and other local news are welcomed. The editorial committee reserves the right to make literary corrections.

Illustrations should always be supplied ready for reproduction. All re-touchings or other operations required will be charged to the author. Line drawings should be drawn in Indian ink. We regret that we must ask authors to pay for making blocks, if this is necessary.

J. W. ARROWSMITH LTD., WINTERSTOKE ROAD

BRISTOL.

### REPORTS FROM SOCIETIES

The SURGICAL CLUB OF SOUTH WEST ENGLAND met in Plymouth on 1st and 2nd May 1964.

Friday, the 1st May was devoted to a demonstration of operations followed by a paper by Mr. Paul A. Bramley on the treatment of fractures of the facial skeleton. In the afternoon there was a discussion on Jaundice and the Surgeons were pleased to welcome a local Physician, Dr. H. M. Leather, and a Pediatrician, Dr. J. M. Montgomery who discussed the medical and children's aspect of this problem.

On Saturday, 2nd May, a number of interesting cases and pathological specimens were demonstrated and later Mr. M. C. T. Reilly described his operation of Sigmoidmyotomy for Diverticulitis. The members of the Club were very interested in this new surgical technique and Mr. Reilly has since lectured in the United States on this subject.

During the Meeting the Surgical Club of South West England made a presentation to Professor Milnes Walker who is, unfortunately, retiring from active membership of the Club. It is to be hoped that he will attend many of the future Meetings but the members felt that his great contribution to the surgery of the South West region whilst he has been Professor of Surgery at Bristol University should be placed on record.

### *Appointments*

### SOUTH WESTERN REGIONAL HOSPITAL BOARD
### CONSULTANTS, SPECIALISTS AND REGISTRARS APPOINTED DURING THE PERIOD JANUARY–JUNE, 1964

### *BRISTOL*

| *Name* | *Appointment* |
|---|---|
| Dallas, N. L., M.A., M.B.B.CH. (Cantab), F.R.C.S. (Ed.) (joint appointment) | Consultant Ophthalmic Surgeon, United Bristol Hospitals/South Western Regional Hospital Board. |
| Griffiths, H. E. D., M.B., B.S. (Lond.), F.R.C.S. (England). | Consultant Orthopaedic Surgeon, Bristol Clinscal Area. |
| McAneny, T. M., B.SC., M.B., B.S. (Lond.), D.A., F.F.A.R.C.S. (Eng.). | Consultant Anaesthetist, Bristol Clinical Area. |
| Davies, J. A. H., M.B., CH.B. (Birmingham), D.OBST., R.C.O.G., D.A., F.F.A.R.C.S. | Senior Anaesthetic Registrar, Cossham–Frenchay Hospital Group. |
| Fisher, A. M., M.A., M.B.B.CHIR. (Cantab), M.R.C.O.G. | Senior Registrar in Obst. and Gynae. Southmead Hospital. |
| Nicholson, I. H., M.B., CH.B. (Edin.), F.R.C.S. | Senior Registrar in Plastic Surgery, Frenchay Hospital. |
| Colton, C. L., M.B., B.S. (Lond.), F.R.C.S. (Eng.). | Surgical Registrar, Cossham–Frenchay Hospital Group. |
| Hinchliffe, Mary K., M.B. B.S. (Lond.), D.C.H., D.OBST., R.C.O.G. | Registrar in Child Psychiatry and Mental Subnormality, Hortham–Brentry Group and Bristol Child Guidance Clinic. |
| Kilby, J. O., M.B., B.S. (Lond.), F.R.C.S. (Eng.). | Surgical Registrar, Southmead Hospital. |
| Thakkar, C., M.B., B.S., M.S. | Registrar in Thoracic Surgery, Frenchay Hospital. |

### *NORTH GLOUCESTERSHIRE*

| | |
|---|---|
| Fraser, I. D., M.D., CH.B. (Bristol). | Consultant Pathologist, Cheltenham General Hospital Group. |
| Nairac, M. L., M.R.C.S., L.R.C.P., D.T.M. & H., D.O.M.S. | Consultant Ophthalmologist, North Gloucestershire Clinical Area. |
| Nicol, A., M.D., CH.B. (Aberdeen). | Consultant Pathologist, North Gloucestershire Clinical Area. |
| Paine, Mary G., M.B., B.S. (Lond.), D.M.R.T. | Consultant Geriatrician, North Gloucestershire Clinical Area. |
| Black, V. H. A., M.B., B.CH. B.A.O., D.P.M. | Senior Registrar in Psychiatry, Horton Road and Coney Hill Hospitals. |
| Hoyland, H. J., M.B., B.CHIR. (Cantab), M.R.C.S., L.R.C.P., D.OBST., R.C.O.G. | Clinical Assistant in Gynaecology, Stroud General Hospital (1 session). |

### *BATH*

| | |
|---|---|
| Craig, J. K., M.B., CH.B (Manchester), F.R.C.S. (Edin. & Eng.), M.R.C.O.G. | Consultant Obstetrician and Gynaecologist, Bath Clinical Area. |
| Wakefield, G., M.B., B.S. (Lond.), M.R.C.P. | Consultant Neurologist, Bath Clinical Area. |
| Yeoman, P. M., M.A., M.D., B.CHIR. (Camb.), F.R.C.S. (Eng.). | Consultant Orthopaedic Surgeon, Bath Clinical Area. |
| Morgan, Eunice V., B.A., M.B., B.CH., B.A.O., D.C.H., D.P.M. | Senior Registrar in Psychiatry, Roundway Hospital, Devizes. |
| Jehan, S. S., M.B., B.S. (Pakistan). | Registrar in Psychiatry, Roundway Hospital. |
| Khan, M. A., M.B., B.S. (West Pakistan), D.T.M. & H. (Lond.). | Medical Registrar, Bath Group of Hospitals. |
| Verma, A. P., M.B., B.S. (Lucknow), D.A. (Bombay). | Orthopaedic Registrar, Bath Clinical Area. |

### *SOUTH SOMERSET*

| *Name* | *Appointment* |
|---|---|
| Laugesan, B. N., M.B., CH.B. (N.Z.), DIP.OBST. (N.Z.). | Surgical Registrar, Taunton and Somerset Hos[illegible] pital. |
| Nair, K. G., M.B., B.S. (Kerala). | Registrar in Psychiatry, Tone Vale Hospital. |
| Rayne, M., M.A., M.D., B.CHIR. (Camb.), M.R.C.P. (Lond.) | Clinical Assistant in Geriatrics, Trinity Hospita[illegible] Taunton (3 sessions). |

### *DEVON AND EXETER*

| *Name* | *Appointment* |
|---|---|
| Shaw, D. B., M.D., B.S. (Lond.), M.R.C.P. (Lond. & Edin.). | Consultant Physician, Devon and Exeter Clinic[illegible] Area. |
| Turner, M. J., M.B., B.CHIR. (Cantab), D.M.R.D., F.F.R., M.R.C.P. (Edin.). | Consultant Radiologist, Torbay Hospital, To[illegible] quay. |
| Wardle, C. J., M.D., B.S. (Lond.), D.P.M. | Consultant in Child Psychiatry, Devon an[illegible] Exeter Clinical Area. |
| Whittard, B. R., M.B., B.S. (Lond.), F.F.A.R.C.S. | Consultant Anaesthetist, Torbay Hospital, To[illegible] quay. |
| Wright, W. B., M.B., CH.B. (Glasgow), M.R.C.P. (Edin.). | Consultant Geriatrician, Devon and Exeter Clin[illegible] cal Area. |
| Verrier-Jones, J., B.A., B.M., B.CH. (Oxon.), M.R.C.P. | Senior Medical Registrar, Royal Devon an[illegible] Exeter Hospital/United Bristol Hospitals. |
| Buse, P. J. De, M.B., B.S. (Lond.), D.C.H. | Registrar in Paediatrics, Royal Devon and Exet[illegible] Hospital/Westminster Hospital. |
| Newton, A. J., M.B., B.S. (Lond.) | Registrar in General Surgery and Orthopaedic[illegible] North Devon Infirmary, Barnstaple. |
| Slater, J. M., M.B., CH.B. (Bristol). | Registrar in Obst. and Gynae., Royal Devon an[illegible] Exeter Hospital/Southmead Hospital, Bristo[illegible] |
| Tresidder, Ann M., M.B.B.S. (Lond.) | Anaesthetic Registrar, Royal Devon and Exet[illegible] Hospital. |
| Bowring Betts, N., M.B., B.CH. (Cantab), F.R.C.S. (Edin.). | Clinical Assistant in E.N.T. Surgery, Bidefo[illegible] (1 session). |
| Whitfield, P. F. G., L.D.S. (Birm.), D.ORTH.R.C.S. (Eng.). | Clinical Assistant in Orthodontics, Royal Dev[illegible] and Exeter Hospital (2 sessions). |

### *PLYMOUTH*

| *Name* | *Appointment* |
|---|---|
| Davidson, G. M., M.B., CH.B. (Glasgow), M.R.C.O.G. | Consultant Obstetrician and Gynaecologist, Pl[illegible] mouth Clinical Area. |
| Gossman, H. H., M.B.B.S. (Durham), F.R.C.S. (Edin.). | Consultant Neurosurgeon, Plymouth Clinic[illegible] Area. |
| Collie, J. A., M.B., CH.B. (Cape Town), D.OBST.R.C.O.G., D.A., M.MED.(Anaes). | Anaesthetic Registrar, Plymouth General Ho[illegible] pital (resigning 30.11.64). |
| Shah, N. M., M.B., B.S., M.S., F.R.C.S. (Edin.). | Orthopaedic Registrar, Mount Gold Orthopaed[illegible] Hospital/Plymouth General Hospital (resig[illegible] ing 31.12.64). |
| Shenoy, K. P., M.B., B.S. (Madras) | Surgical Registrar, Plymouth General Hospital[illegible] |
| Webb, M. A. H., M.R.C.S., L.R.C.P. | Registrar in E.N.T. Surgery, Plymouth Gener[illegible] Hospital. |

### *WEST CORNWALL*

| *Name* | *Appointment* |
|---|---|
| Murrell, J. S., M.B., B.S. (Lond.), M.R.C.P. (Edin.). | Consultant Pathologist, West Cornwall Clinic[illegible] Area. |
| Attalla, S. M., M.B., CH.B. (Egypt). | Orthopaedic Registrar, Royal Cornwall Infirmar[illegible] Truro. |
| Chawla, J. C., M.B.B.S. (Osmania). | Surgical Registrar, Royal Cornwall Infirmar[illegible] Truro. |

Printe[illegible]

J. W. Arrowsmith Ltd., Bri[illegible]

